# Cell-Based and Selected Cell-Free Therapies for Myocardial Infarction: How Do They Compare to the Current Treatment Options?

**DOI:** 10.3390/ijms231810314

**Published:** 2022-09-07

**Authors:** Mária Csöbönyeiová, Nikoleta Beerová, Martin Klein, Michaela Debreová-Čeháková, Ľuboš Danišovič

**Affiliations:** 1 National Institute of Rheumatic Diseases, Nábrežie I. Krasku 4, 921 12 Piešťany, Slovakia; 2 Institute of Histology and Embryology, Faculty of Medicine, Comenius University, Sasinkova 4, 811 08 Bratislava, Slovakia; 3 Institute of Medical Biology, Genetics and Clinical Genetics, Faculty of Medicine, Comenius University, Sasinkova 4, 811 08 Bratislava, Slovakia

**Keywords:** myocardial infarction, heart regeneration, stem cells, exosomes

## Abstract

Because of cardiomyocyte death or dysfunction frequently caused by myocardial infarction (MI), heart failure is a leading cause of morbidity and mortality in modern society. Paradoxically, only limited and non-curative therapies for heart failure or MI are currently available. As a result, over the past two decades research has focused on developing cell-based approaches promoting the regeneration of infarcted tissue. Cell-based therapies for myocardial regeneration include powerful candidates, such as multipotent stem cells (mesenchymal stem cells (MSCs), bone-marrow-derived stem cells, endothelial progenitor cells, and hematopoietic stem cells) and induced pluripotent stem cells (iPSCs). These possess unique properties, such as potency to differentiate into desired cell types, proliferation capacity, and patient specificity. Preclinical and clinical studies have demonstrated modest improvement in the myocardial regeneration and reduced infarcted areas upon transplantation of pluripotent or multipotent stem cells. Another cell population that need to be considered as a potential source for cardiac regeneration are telocytes found in different organs, including the heart. Their therapeutic effect has been studied in various heart pathologies, such as MI, arrhythmias, or atrial amyloidosis. The most recent cell-free therapeutic tool relies on the cardioprotective effect of complex cargo carried by small membrane-bound vesicles—exosomes—released from stem cells via exocytosis. The MSC/iPSC-derived exosomes could be considered a novel exosome-based therapy for cardiovascular diseases thanks to their unique content. There are also other cell-free approaches, e.g., gene therapy, or acellular cardiac patches. Therefore, our review provides the most recent insights into the novel strategies for myocardial repair based on the regenerative potential of different cell types and cell-free approaches.

## 1. Introduction

Ischemic heart disease (IHD) is a leading cause of death worldwide. In addition, referred to as coronary artery disease (CAD) and atherosclerotic cardiovascular disease (CVD), it manifests clinically as myocardial infarction (MI) and ischemic cardiomyopathy [1].

MI, commonly known as the “heart attack,” is caused by decreased or complete cessation of blood flow to a portion of the myocardium. In most cases, the occlusion results from plaque rupture with subsequent thrombosis [2]. The definition of MI was formally redefined in 2000 by the European Society of Cardiology and the American College of Cardiology according to myocardial injury corroborated by biomarkers with particular emphasis on troponin. This definition was updated in 2007 to reflect the progress made in understanding assays. It was again centered around troponin. It was decided that with rare exceptions the diagnosis cannot be made in the absence of elevated biomarkers of cardiac injury [3]. Each year, the American Heart Association publishes its annual update of heart disease and stroke statistics. After analyzing the latest two updates of 2021 providing 2019 statistics [4] and 2022 providing 2020 statistics [5], a worrisome trend can be observed. In 2019, approximately 18.6 million people died due to CVD around the globe, compared to 19.05 million in 2020. Similarly, the crude prevalence of CVD in 2019 was 523.2 million cases, while in 2020, this figure rose to 607.64 million. Speaking of CVD epidemiology with respect to individual continents, both years’ statistics show that the highest mortality rates are in Eastern Europe and Central Asia. From all deaths due to noncommunicable diseases, CVD is estimated to account for about 37%. According to WHO, from all the 2019 deaths attributed to CVD, 85% were due to heart attack and stroke [6]. According to a 2020 study, IHD affects around 126 million individuals (1655 per 100,000), which is approximately 1.72% of the world’s population. The current prevalence rate of 1655 per 100,000 population is expected to exceed 1845 by 2030. Corresponding to the above-discussed mortality rates of CVD in general, the highest prevalence of IHD is also observed in Eastern European countries [1]. Fortunately, thanks to novel evidence-based therapies and lifestyle changes, the mortality rate of IHD has been reduced in recent decades [7]. Nevertheless, the standard therapy of MI is incapable of repairing the ischemic damage to the myocardial tissue resulting in limited physical activity, lifetime use of medication, and a higher risk of heart failure recurrence [8]. Therefore, there has been an intense search for alternative treatment options focusing mainly on cell-based therapy and tissue engineering. This review article provides an overview of current treatment strategies, with the main emphasis on rapidly developing future treatment options, specifically cell-based and cell-free therapies.

## 2. Myocardial Infarction (MI)—Pathology, Clinical Presentation, Diagnostics, and Types

### 2.1. Etiopathogenesis

The pathological definition of MI is based on prolonged myocyte ischemia leading to myocardial cell death. Myocardial ischemia may result either from increased demand for oxygen, decreased oxygen supply to the myocardium, or both. After 10–15 min of coronary occlusion, myocardial necrosis ensues. Unlike, e.g., zebrafish or even prenatal/neonatal mice whose hearts can regenerate quite well, necrosis of adult mammalian myocardium is a serious condition since cardiomyocytes (CMs) as terminally differentiated cells have weak regenerative abilities [9]. The first ultrastructural changes to observe are diminished cellular glycogen, relaxed myofibrils, and sarcolemmal disruption. Electron microscopy shows mitochondrial abnormalities as early as 10 min after coronary occlusion, and these changes are progressive [10]. Over several hours, necrosis spreads from subendocardial to subepicardial parts of the myocardium. After 40 min of occlusion, necrosis is 38% complete; at 3 h, it is 57% complete; at 6 h, 71% complete; and at 24 h, 85% complete [11]. The duration may be extended by increased collateral flow, reduced myocardial oxygen consumption, and intermittent occlusion/reperfusion, which can precondition the heart. The timely implementation of reperfusion therapy might reduce ischemic injury of the myocardium [12].

### 2.2. Clinical Presentation and Diagnostics

The term acute MI (AMI) should be used when there is an acute myocardial injury with clinical evidence of acute myocardial ischemia and with detection of a rise and/or fall of cardiac troponin (cTn) values with at least one value above the 99th percentile upper reference limit and at least one of the following: (1) symptoms of myocardial ischemia, (2) new ischemic electrocardiographic (ECG) changes, (3) development of pathological Q waves, (4) imaging evidence of new loss of viable myocardium or new regional wall motion abnormality in a pattern consistent with an ischemic etiology, and (5) identification of a coronary thrombus by angiography or autopsy (not for type 2 or 3 MIs). Possible ischemic symptoms include various combinations of chest, upper extremity, mandibular, or epigastric discomfort during exertion or at rest or an ischemic equivalent such as dyspnea or fatigue. Often, the discomfort is diffuse, not localized, positional, or affected by movement. MI may present with atypical symptoms such as palpitations, cardiac arrest, or there can be no symptoms whatsoever [10]. Supposed myocardial ischemia presents clinically and/or is detected by ECG changes together with myocardial injury manifested by a rising and/or falling pattern of cTn values. In that case, a diagnosis of AMI is appropriate [13].

### 2.3. Types of Myocardial Infarction (MI)

The ST-elevation MI (STEMI) vs. non-ST-elevation MI (NSTEMI) paradigm is based on the randomized controlled thrombolytic trials in the 1980s and 1990s in which the outcome measure was mortality. This classification does not include angiographic coronary occlusion [14]. STEMI is characterized by new ST-segment elevations in two contiguous leads or new bundle branch blocks with ischemic repolarization patterns. In contrast, patients without ST-segment elevation are usually diagnosed with NSTEMI. The categories of patients with STEMI, NSTEMI, or unstable angina are customarily included in the concept of acute coronary syndrome. In addition to these categories, MI may be classified into various types based on pathological, clinical, and prognostic differences, as well as different treatment strategies [10].

In 2018, the fourth universal definition of MI was published. Type 1 AMI corresponds to acute thrombosis on a ruptured or eroded atherosclerotic plaque, and type 2 AMI occurs based on the mismatch between oxygen supply and demand. Type 3 AMI represents sudden cardiac death of presumed ischemic origin and AMI detected by autopsy. Type 4 and 5 AMI are related to coronary procedures (stent or scaffold thrombosis, percutaneous coronary intervention (PCI), or coronary artery bypass grafting (CABG) [15].

## 3. Current Therapies for MI

Over the years, reperfusion has been shown as the most effective therapy against ischemic damage during STEMI [12]. The main focus is on the expeditious opening of the affected vessel within the treatment algorithm of AMI [15]. The recanalization of the occluded coronary artery restores perfusion and prevents myocardial necrosis. Standard methods to ensure reperfusion include drug therapy, thrombolytic therapy, PCI, and CABG surgery [16].

### 3.1. Drug Therapies

Conventional drug therapy algorithms include:

- Antithrombotic drugs,
- Β-receptor blockers,
- Angiotensin-converting enzyme inhibitors,
- Angiotensin receptor blockers and statins.

This drug algorithm is used to prevent left ventricle remodeling and to avoid the progression of the atherosclerotic process and the recurrence of MI [17].

### 3.2. Thrombolytic Therapy

At present, the main approach to the treatment of MI is thrombolytic therapy. Its main goal is to recanalize the occluded coronary artery and restore the perfusion within the shortest time period possible. Their mechanism of action is dissolving the thrombus in the affected artery, achieving reperfusion. The effect is reduced infarct size, preserved left ventricular function, and improved survival. Currently, the most commonly used thrombolytic drugs are streptokinase, urokinase, and tissue-type plasminogen activators. However, this therapeutic approach has side effects, including unwanted bleeding, from minor to life-threatening intracerebral hemorrhage [18].

### 3.3. Percutaneous Coronary Intervention

Considering the limitations of thrombolytic therapy, PCI has gradually become the principal choice of restoring coronary perfusion. Compared with medical treatment, percutaneous revascularization is a reasonable option to improve survival [19]. This minimally invasive procedure effectively secures blood flow restoration and recovers the heart muscle function by inserting a special catheter into a blood vessel and inflating the narrowed area of the coronary artery [20]. Compared with thrombolytic therapy, PCI removes the thrombus, and the reperfusion rate after the intervention is 95% to 99%. However, PCI carries possible risks, such as bleeding or infection at the catheter insertion site, allergic reaction to the contrast dye used, blood clot within the treated blood vessel, rupture of the coronary artery, or complete closure of the coronary artery [21,22]. Another very serious potential complication of PCI is reperfusion injury. It is defined as a myocardial and vascular injury as a direct consequence of blood flow restoration that can even lead to death (lethal reperfusion injury). Therefore, PCI should always be performed with regard to limiting the risk of reperfusion injury which by itself requires a complex approach with an uncertain result [23].

### 3.4. Coronary Artery Bypass Grafting

Commonly used treatment approaches include CABG, an effective surgical treatment of coronary heart disease and myocardial ischemia. It can effectively relieve symptoms and is also a method of choice when treating restenosis and acute complications in patients after PCI [24].

Current guidelines recommend early mechanical revascularization with PCI as the first therapeutic option for AMI treatment in the general population [15]. CABG usually assumes a time delay. Because in AMI, “time is muscle”, CABG is currently used in only 5% of AMI patients. It is reserved for patients with complex CAD as a three-vessel disease, left main stenosis, or stenosis of the proximal left anterior descending artery [25].

Although these treatments can to some extent relieve the symptoms of myocardial ischemia, they have limited capabilities of fully protecting the tissue by preventing the spread of injury at the border zone and most importantly, they do not address the issue of myocardial regeneration by means of regrowing new CMs. Myocardial regeneration is undoubtedly the best solution to the clinical problems of MI treatment. Therefore, in recent years, myocardial regeneration has become the hotspot of cardiovascular research [26]. Nevertheless, it is still considered a theoretical approach since a satisfactory clinical application has not yet been achieved.

## 4. Cell-Based Therapies for MI

A promising alternative to existing medical and interventional treatments is cell-based therapies using the advantages of multipotent and pluripotent stem cells. These cells can differentiate into cardiac progenitor cells (CPCs) or CMs, which can form new contractile tissue and prevent adverse remodeling of the post-MI myocardium [27,28,29]. Nowadays, cell transplantation is the most intensively studied strategy, with numerous successful outcomes in animal in vivo and in vitro models [30,31]. The regenerative approach has not just preventive effect but also curative in terms of new CM formation and regeneration of blood vessels. Moreover, several clinical trials have already tested myocardial cell grafting on patients with MI and CAD [32,33]. Even so, before routine clinical application of stem cells, it is necessary to resolve safety issues regarding the risk of oncogenic transformation, inefficient delivery to the site of injury, problems with cell engraftment due to the lack of microvasculature, cell senescence, and the electric coupling of transplanted CMs within the myocardium. There is also another limitation causing poor cell graft viability in terms of cell adhesion loss driven by the mechanism of anoikis [34,35,36]. However, the solution to the mentioned obstacles may be the coordination of cell-based technologies with rapidly developing bioengineering strategies, such as 3D bioprinted cardiac microtissues or patches [37,38].

### 4.1. Mesenchymal Stem Cell-Based Therapy for MI

The primary candidates for reconstruction and functional improvement of damaged myocardium are adult stem cells/multipotent mesenchymal stem cells (MSCs) isolated from bone marrow, heart tissue, adipose tissue, circulating blood, endothelium, and umbilical cord [39,40,41]. In clinical trials focused on MSCs-based therapy, bone marrow-derived MSCs (BM-MSCs) are the first choice thanks to their autologous nature and standardized isolation methods. However, there are also other types of stem cells whose cardiac regenerative potential has been investigated, including menstrual blood-derived endometrial stem cells (MenSCs) [42] or fetal membrane-derived MSCs (Fm-MSCs) [43,44]. It is well known that MSCs possess anti-fibrotic, immunomodulatory, anti-apoptotic, anti-oxidant, and pro-angiogenic features. Nonetheless, it was shown that the beneficial effect of different types of MSCs is modest due to their primarily paracrine effect. Moreover, the optimal way of their transplantation and the proper number of cells is required for a satisfactory therapeutic outcome [45,46,47]. The alternative to the cells mentioned above are embryonic stem cells (ESCs) and induced pluripotent stem cells (iPSCs), which can give rise to all cells of our body. Due to ethical concerns and a considerable risk of rejection, only a few studies have investigated the feasibility of ESC-derived CMs for heart regeneration [48,49,50,51]. On the other hand, developing iPSC technology has dramatically boosted cardiac cell-based therapy since iPSCs have unlimited capacity to generate clinically relevant cell types, including CMs. Moreover, cells differentiated from iPSCs are patient-specific, so there is almost no risk of immunological rejection. At the same time, the clinical use of iPSCs is impeded by the risk of genetic and epigenetic abnormalities and eventual teratoma formation; therefore, it is essential to fine-tune several factors, such as reprogramming techniques, cell purification, and optimal transplantation method [47,52].

The most widely studied stem cells for heart regeneration are **BM-MSCs**. Numerous studies have proved the beneficial effect of BM-MSCs on regenerating myocardium in animal models in vivo and in vitro [53,54,55,56]. For instance, Karpov et al. investigated the effect of BM-MSCs and adipose tissue-derived stem cells (AD-MSCs) intramyocardial transplantation on peri-infarcted tissue in the Wistar rat model of myocardial ischemia-reperfusion. The isolated hearts were examined two weeks after stem cell injection. The hearts injected with BM-MSCs displayed better preservation of the left ventricle contractility during ischemia and notably smaller infarct areas than those treated with AD-MSCs [57]. Moreover, the therapeutic use of BM-MSCs is preferred over AD-MSCs thanks to their pro-angiogenic and immunomodulatory properties [58]. Lim et al. came up with the idea to use macrophages and BM-MSCs to enhance cardiac repair, considering that anti-inflammatory macrophages are mainly involved in the healing of infarcted myocardium [59]. Furthermore, it has been hypothesized that the regenerative effect of BM-MSCs is under the influence of macrophages. To test this, Ben-Mordechai et al. prepared a mixture of BM-MSCs and anti-inflammatory macrophages isolated and differentiated from bone marrow. This mixture was injected into the peri-infarct area of the left ventricular myocardium of a mice MI model. After two weeks, histological and immunohistochemical analyses showed notable improvement in the structure and function of the infarcted myocardium although the authors noted some limitations of their study, such as a lack of in vivo verification of BM-MSCs differentiation into CMs and insufficient histological evaluation of interstitial collagen fibers deposition [60]. Currently, it is believed that BM-MSCs probably do not differentiate into functional CMs within damaged tissue; rather, their paracrine effect helps in regeneration [61,62,63].

Intending to examine whether transplantation of BM-MSCs can improve the left ventricular function in patients with AMI, Lee et al. conducted a randomized, open-label pilot study. The study enrolled 80 patients who randomly received an intracoronary administration of autologous BM-MSCs into the infarct-related artery. The authors studied left ventricular ejection fraction (LVEF) changes and the possible incidence of treatment-related adverse effects during the follow-up period. The clinical and functional analyses were performed on the first, second, and sixth months after transplantation. The procedures involved coronary angiography, electrocardiogram-gated single-photon emission computed tomography, and echocardiography. According to the results, the LVEF displayed only modest improvement compared with the control group. Authors hypothesized whether the slight increase in systolic function is not only statistical. The procedure’s safety was sufficient, with almost no serious adverse events [32].

Another multi-center randomized, double-blind, and placebo-controlled trial was focused on the therapeutic effect of intracoronary delivery of autologous BM-MSCs at 1–7 days post-AMI. A total of 100 patients were randomized in a ratio of 1:1 to receive an intracoronary infusion of BM-MSCs or a placebo. The LVEF was observed between baseline and one year by advanced cardiac imaging; however, only a small non-significant improvement was detected compared to placebo. On the other hand, the myocardial salvage index was considerably increased, indicating a positive effect on myocardial remodeling [64]. In the most recent paper of the same research group, the authors published a five-year follow-up focused on major adverse cardiac events, such as recurrent MI, all coronary revascularization, and overall cause of death after the intracoronary delivery of BM-MSCs. Contrary to the first-year outcome, current results showed no difference or improvement compared with the placebo group [65].

Similarly, the results of the latest innovative randomized, single-blind clinical trial, in which enrolled patients with ST-segment elevation MI underwent autologous BM-MSCs therapy, did not meet the expectations. Patients received BM-MSCs injection via percutaneous coronary artery perfusion, and after that, the researchers evaluated the changes in myocardial metabolic activity at the 6th month post-transplantation and at the 12th month, the changes in LVEF. Likewise, they analyzed mortality and adverse effects related to the transplantation. Results showed no significant differences in the metabolic imaging defect score and no improvement in LVEF between the BM-MSCs transplantation patients and the control group. Adverse cardiovascular events were also similar in both groups [33]. The contradictory results of other published clinical trials have raised many questions about the limitations of autologous BM-MSCs transplantation in terms of the optimal way of cell administration, the dose of transplanted cells, duration of cell cultivation, and appropriate time of cell transplantation [66,67,68,69]. Moreover, according to recent studies, the MSCs may not be immune-privileged; therefore, their use in the clinic can significantly increase the risk of adverse effects [70].

Other MSCs involved in the randomized, controlled trial conducted by Gao et al. research group were MSCs-derived from the Wharton’s jelly (**WJ-MSCs**). In this multicentered trial, 116 patients were enrolled suffering from an acute ST-elevation MI. Randomly chosen patients received intracoronary infusion with WJ-MSCs into the infarct artery. The main end point was to evaluate the safety and LVEF within 18 months and myocardial viability and perfusion of the infarcted area within 4 months after the surgery. In all setup goals, the group of WJ-MSCs showed significantly increased parameters over the placebo group, with no safety issues at the same time. Based on these encouraging results, the authors suggested running additional clinical trials to confirm that the WJ-MSCs could represent an alternative to BM-MSCs for myocardial regeneration [40].

The umbilical cord is a rich source of stem and progenitor cells, which have been used to treat a wide range of blood-related diseases including leukemia or anemia. **Umbilical cord stem cells (UCB-SCs)** have several advantages over the BM-MSCs, such as higher proliferation, easy harvesting, decreased risk of unsuccessful grafting, enhancement of angiogenesis, and anti-fibrotic effects. Moreover, they have lower immunogenicity [71,72]. Therefore, it is not surprising that UCB-SCs are considered one of the potential cell sources for MI therapy. Based on the successful studies on animal MI models, the team of Bartolucci performed a clinical trial (RIMECARD), and for the first time, they evaluated the efficacy and safety of intravenous infusion of allogenic UCB-SCs in patients with heart failure and reduced ejection fraction. A group of patients receiving UCB-SCs displayed notable improvements in LVEF at 3, 6, and 12 months of follow-up but no significant reduction in left ventricular end-systolic volume (LVESV) and end-diastolic volume (LVEDV) [73]. However, the comparison of paracrine factors between UCB-SCs and BM-MSCs in vitro resulted in a considerable advantage for UCB-SCs, particularly in high expression of hepatocyte growth factor, which is believed to be one of the most important factors promoting angiogenesis and decreasing fibrosis in the infarcted area [74]. Overall, the intravenous infusion therapy was safe with no humoral response. Yet, the study’s major limitation is the small number of participants; therefore, the authors suggested further research through extensive clinical trials. Umbilical cord stroma (UCS)-MSCs were also tested in a clinical trial completed in 2018. The results were a possible positive effect in scar tissue reduction and restoration of ventricular wall function, thus showing possible efficacy in the management of chronic ischemic cardiomyopathy [75]. MSCs harvested from the umbilical cord were also tested in a clinical trial concluded in 2022. The final data are yet to be collected and evaluated [76]. Another approach to clinical trial was the use of cardiosphere-derived cells (CDCs)—CPCs with disease-modifying bioactivity. Although safe, this approach failed to reduce scar size compared to placebo at 6 months. On the other hand, reductions in LVEDV and LVESV may indicate disease-modifying capacity of CDCs [77].

A promising source of multipotent stem cells for cardiac therapy seems to also be those obtained from menstrual blood—**blood-derived endometrial stem cells (MenSCs)**. They can be periodically and non-invasively collected with the ability to transdifferentiate into CMs with higher efficacy than BM-MSCs [42,78]. For instance, Hida et al. transplanted MenSCs in the MI area of nude rat models resulting in improved cardiac function and decreased fibrosis, not only due to the induced neovascularization and anti-apoptotic effect but also thanks to the massive cardiogenic transdifferentiation of MenSCs. Despite the superior capacity of MenSCs, their use is still far from clinical practice and requires further research on the therapeutic mechanism, optimal cell delivery methods, and cell doses [79].

Some researchers focus on **amniotic membrane-derived stem cells (FM-MSCs) as an alternative to BM-MSCs** in myocardium repair. Cells of the amniotic membrane have the potential to transdifferentiate into multiple cell lineages, and more importantly, they display low immunogenicity and high histocompatibility with no risk of teratoma formation in vivo [80]. Tsuji et al. examined whether xenografted human FM-MSCs could be used as an allograftable and immunologically tolerable cell source for cardiac repair after MI. Researchers transplanted human FM-MSCs into the myocardium of MI nude and the Wistar rat model. The effect of FM-MSCs on cardiac function was examined after 2 weeks post-surgery. According to obtained results, there was a significant increase in left ventricular fractional shortening and a notable decrease in the fibrosis area. Moreover, at 4 weeks after transplantation, there was no need to use any immunosuppressants [43]. Ishakane et al. transplanted allogenic FM-MSCs sheets into the scarred myocardium of the MI rat model and compared their therapeutic potential with transplanted autologous BM-MSCs sheets. Both stem cell transplants had significantly improved cardiac function, robust angiogenesis, and reduced myocardial fibrosis. Despite that, the study possesses some limitations, such as the poor survival time of transplanted cells and their low differentiation rate [44]. More recent studies on animal models showed similar results in terms of improved heart performance and a decrease in fibrosis [81,82]. On top of that, Takov et al. demonstrated that FM-MSCs exhibit pro-angiogenic and anti-apoptotic effects in MI through the secretion of small extracellular vesicles [83]. Nevertheless, several limitations hamper the use of FM-MSCs in clinics, including insufficient cell quality, possible cross-contamination, lack of standardized isolation and purification protocols, and the fact that most studies were conducted on rat models; therefore, allogenic tolerability in humans is unclear.

Taken together, there are a number of preclinical and clinical trials focus on myocardial repair after MI by different types of MSCs; however, the outcome from these trials remains disappointing, with relatively similar modest benefits for MI regeneration therapy [84,85,86]. Selected clinical trials are summarized in Table 1.

A major issue is the short lifespan and persistence of transplanted cells; therefore, there are attempts to enhance their engraftment and persistence via different genetic modifications, such as overexpression of anti-apoptotic and pro-survival factors Akt and Pim1 [102,103,104,105]. Ebeid et al. investigated the relationship between the cardioprotective kinase Pim1 and the transforming growth factor-beta (TGFβ) pathway, which regulates telomere length in CPCs. The authors revealed the process that delays cellular senescence. Pim1 inhibits the phosphorylation of the TGFβ pathway downstream effectors (Smad 2 and Smad3), thus preventing telomerase repression. This critical finding can contribute to implementing novel targets to enhance the lifespan of grafted cells [106]. Another extensively investigated way to boost the therapeutic effect of transplanted cells is combining different stem cell populations and applying preconditioned media [107].

### 4.2. Induced Pluripotent Stem Cell (iPSC)-Based Therapy for MI

The field of regenerative medicine was revolutionized with the tremendous discovery of reprogramming technology leading to the generation of iPSCs by the team of Nobel prize winner prof. Yamanaka [108]. The capability to reprogram an adult somatic cell toward a pluripotent stem cell by a defined cocktail of reprogramming factors (Oct3/4, Sox2, c-Myc, and Klf4) has started a new era of stem cell-based therapy. The iPSCs display characteristics similar to ESCs, and their use bypasses the ethical controversy linked with the ESCs. Thanks to the pluripotent nature of iPSCs, they can give rise to cell lineages of all three germ layers, including CMs. Moreover, they are patient-specific, thus eliminating immune rejection. The success of CM generation depends particularly on the reprogramming, differentiation, and purification techniques. From the initial reprogramming using retroviruses, the researchers have developed a number of safer and more effective methods, such as nonintegrating Sendai virus, synthetic plasmids, miRNA, recombinant proteins, Cre-lox system, and small molecules [109,110]. Regarding the differentiation of iPSCs into CMs, the most widely used basic approaches are 3D embryoid body formation, 2D monolayer culture, co-culture system, and manipulation of the canonical Wnt pathways by small molecules [111,112,113]. More detailed step-wise differentiation protocols are discussed in our previous work [114]. Nevertheless, the methods mentioned above are not optimal due to the eventual generation of heterogeneous cell populations, including immature forms of iPSC-derived CMs (iPSC-CMs) or non-differentiated cells, whose transplantation can increase the risk of tumor formation [115]. To select only the fully differentiated iPSC-CMs, it is necessary to apply purification techniques, of which the most effective seem to be a non-genetic targeted antibody-cell sorting resulting in >99% cell purity or a newly established method using mitochondria-specific viable fluorescent dyes [110,116]. Another selection strategy is cultivating differentiated cells in a low-glucose, high lactate medium with a purification efficacy of around 98% [117].

Over the past two decades, the iPSC-CMs generation, thanks to the improvement of differentiation and purification techniques, has opened the door for clinical applications. The underlying mechanism of MI and the therapeutic effect of iPSC-CMs have been demonstrated in several small and large animal models of MI, such as mice [118,119], rats [120,121], and pigs [121,122,123]. A significant study published by Chong et al. regarding the use of ESC-CMs in a non-human-primate model (pigtail macaque) of myocardial ischemia-reperfusion resulted in remuscularization of heart tissue but incomplete cell maturation [50]. The research was followed by the study of Shiba et al. in MI cynomolgus monkey models using iPSC-CMs, in which iPSC-CMs transplantation improved cardiac contractile function; however, the incidence of post-transplant arrhythmias was considerably higher [124]. During the past 4 years, there has been tremendous progress toward the clinical use of iPSCs for treating damaged heart tissue. The team of professor Sawa from Osaka University in Japan has performed the world’s first transplant of 100 million iPSC-CMs seeded on degradable sheets into 10 patients suffering from ischemic cardiomyopathy. It is expected that sheets will be attached to the surfaces of the patient’s heart and, via cell secretion, will regenerate the blood vessels and improve cardiac function [125]. Another research group from Nanjing University Medical school in China has started to evaluate the safety, feasibility, and efficacy of the clinical application of iPSCs technology on patients with heart failure in their ongoing study [126]. The single-center phase I/IIa clinical trial is dose-escalation and placebo-controlled. The six enrolled participants will be treated with human allogenic iPSC-CMs from healthy donors. The researchers plan to inject 200 million human iPSC-CMs suspended in a medium directly into the myocardium during coronary artery bypass surgery. The primary outcome measures will focus on the incidence of sustained ventricular arrhythmias and newly formed tumors. Since the study is planned to be completed by the middle of 2023, the results have not been published yet. Nevertheless, the cardiac surgeon who performed the transplantation reported very satisfactory outcomes in the first two patients who underwent the surgery [127]. This year in April, a multi-center, open-label, non-randomized phase I/II clinical trial started in Japan, intending to transplant iPSC-CMs spheroids in 10 patients with severe heart failure. Afterwards, the efficacy, tolerability, and safety of iPSC-CMs spheroids needle transplantation will be evaluated for 26 weeks (LAPiS Study) [101].

### 4.3. iPSC-Derived Cardiomyocyte Cardiac Patches and Bioprinting

In most preclinical and clinical experiments, the delivery method of iPSC-CMs has often been performed via intracoronary infusion or direct myocardial injection; however, this approach proved to be less effective, resulting in a low engraftment rate and poor survival of transplanted cells. Thanks to the fast development of cardiac tissue engineering techniques in recent years, the opportunity to use engineered tissues, such as cardiac patches, has become an attractive delivery method for iPSC-CMs. Various therapeutic agents, such as iPSC-CMs, MSCs, myoblasts, and bioactive molecules, could be incorporated within cardiac patches [128,129,130]. Numerous studies have investigated the efficacy of 3D-engineered cardiac patches made from various biomaterials, including hydrogels and decellularized scaffolds [131,132,133,134,135,136]. However, at the same time, the ideal material for cardiac patch construction is still out of reach. In vivo studies using engineered cardiac patches on small and large MI animal models have shown an overall reduction in the scar size, increased wall thickness, and improvement in LVEF [137,138]. For instance, a work published by Wendel et al. examined the regenerative efficacy of engineered cardiac patches made from iPSC-CMs and pericytes entrapped in fibrin gel on a nude rat model of AMI. The choice to add pericytes was based on their ability to induce fibrin gel compaction and support microvascularization of the patch. The cardiac patches were directly transplanted into the injured myocardium, and 4 weeks following transplantation, echocardiography and histological analyses were performed. Results displayed satisfactory viability and proliferation of iPSC-CMs, leading to a reduction of infarcted area and more significant fractional shortening than the control group. However, the considerable limitation of this study was the small sample size and the absence of long-term observation [139]. Gao et al. fabricated a novel clinically relevant size and thickness type of cardiac-muscle patches made from trilineage cardiac cells derived from iPSCs (CMs; smooth muscle cells, endothelial cells) seeded in a 3D fibrin scaffold. The efficacy of patches was examined on a porcine model of MI. Following scientific evaluations (MRI, hemodynamic analyses) revealed a high engraftment rate at week 4 after transplantation and significant improvement in LVEF, myocardial hypertrophy, reduced apoptosis, and infarction size. Even though the results are optimistic, the observation period was again too short [130].

More recently, decellularized materials are becoming an attractive source of natural scaffolds, replacing synthetic scaffolds and biopolymers, which lack complex biochemical properties and 3D structure. Several successful attempts have been made to engineer cardiac patches from decellularized ventricular structures or whole hearts. The advantage of such cardiac patches is that they retain the anisotropic architecture of the heart and essential components of the extracellular matrix (ECM) [140,141,142].

In a pioneer experiment published by Lu et al., the researchers repopulated decellularized mouse hearts with iPSC-derived cardiovascular progenitors, which subsequently proliferated and differentiated into mature CMs, smooth muscle cells, and endothelial cells. After 20 days, the spontaneous contraction of the engineered heart tissue was detected. However, the repopulated areas of the heart were not uniform in cell distribution [143]. Guytte reported the recellularization of post-mortem human acellular hearts by non-transgenic human iPSC-CMs. After 12 days, seeded heart scaffolds cultured in a biomimetic bioreactor were metabolically active exhibiting contractile function and responding to electrical stimuli. Nevertheless, complete heart recellularization was not achieved [144]. Wang et al. fabricated functional cardiac patches from pieces of decellularized natural heart ECM seeded with human iPSC-CMs and fibroblasts. The patches exhibited uniform beating activity and electrical physiology in vitro, similar to normal human CMs. Moreover, the implantation of patches on the damage site of an AMI rat model improved heart function even though some disadvantages of such engineered heart constructs include the requirement of healthy donor hearts, the lack of optimal seeding method, and possible immune reaction [145].

Jiang et al. generated the bioengineered cardiac patches made from the decellularized placenta and iPSC-CMs to evaluate the impact of this natural scaffold material on the engraftment and survival of transplanted cells in a rat model of MI. The histological and protein analyses were performed 4 weeks post-transplantation. Results revealed that decellularized placenta retain considerable growth and pro-angiogenic factors, augmenting the iPSC-CMs survival and maturation. Moreover, the transplanted cardiac patches promoted neovascularization, reduced the infarcted area’s size, and improved LVEF. This research was performed only in a small rat model; therefore, it would be beneficial to complete it in larger animal models or non-human primates [135].

3D bioprinting technologies are considered an alternative to decellularization methods, allowing for printing the high-resolution tissues or scaffolds with complex architecture and organized cell placement. There have been attempts to fabricate 3D printed cardiac patches from natural and synthetic hydrogels, decellularized ECM (dECM)-based bioinks, or even biomaterial-free bioprinted patches [37,146,147]. Natural hydrogel-based materials have several advantages over the synthetic ones in terms of their ability to mimic specific tissue microenvironments and support cell adhesion and proliferation. A powerful type of biomaterials represents dECM-based bioinks. Encouraging results using dECM-based 3D bioprinted cardiac patches were reported by several authors. For instance, the porcine heart-derived dECM-based hydrogel constructed by Traverse et al. [142], and similarly Pati et al. [148] and Jang et al. [149], bioprinted cardiac tissue-derived dECM hydrogels.

The 3D bioprinted cardiac patches without biomaterial were developed by Ong et al. The investigators 3D bioprinted cardiac patches from multicellular spheroids composed of human iPSC-CMS, human adult ventricular cardiac fibroblasts, and human umbilical vein endothelial cells. In vitro, electrophysiological analyses showed uniform electrical conduction with higher conduction velocities. In vivo implantation of cardiac patches onto nude rats’ hearts revealed high cell density and rudimentary blood vessels formation [146]. Likewise, Yeung et al. used a similar way of cellular spheroids 3D bioprinting to create cardiac patches. Patches were transplanted into a rat MI model, and their therapeutic potential was examined 4 weeks after implantation. Compared with the control group, the average vessel counts in damaged areas were higher and scar areas notably smaller; furthermore, the researchers also detected an improvement in heart function [37]. Nevertheless, despite the undeniable outcomes of studies mentioned above, 3D printed cardiac patches are far from clinical use because of certain limitations regarding the manufacturing techniques, the small size of the implant, and its low mechanical strength [150,151].

A step forward in scaffold engineering was made by the team of Cui et al., who printed a 4D physiologically adaptable cardiac patch by beam-scanning stereolithography using a gelatin-based printable ink. This specific printing technique allows the printing of a highly stretchable microfabricated tissue construct, increasing its mechanical tolerance. Moreover, the triculture of printed tissue with iPSC-CMs, MSCs, and endothelial cells reproduced the anisotropy of elastic epicardial fibers and enhanced vascularization of the cardiac patch. After 7 days of co-culturing, the significant contraction of iPSC-CMs and higher density of capillary-like endothelial cells distribution were observed. In vivo evaluation was made on a murine model of ischemia-reperfusion injury. The patches were directly placed over the infarcted tissue area of the heart’s epicardium. A total of 10 weeks post-surgery, the histological analyses showed a smaller size of the infarcted area, and cardiac magnetic resonance imaging revealed contraction and relaxation of the cardiac patch along with the mouse’s heartbeat together with prominent blood perfusion. Similar results were also observed 4 months after implantation. The cardiac patch was vascularized and firmly attached to the heart tissue; however, integrated cells exhibited immature 3D sarcomeric organization [134]. Undoubtedly, developing advanced bioprinting techniques will enable the generation of more suitable engineered cardiac tissue ready for clinical implementation; nevertheless, before this last step, long-term experiments with precise analyses in preclinical studies must be conducted [152].

### 4.4. Potential of Telocytes in the Therapy of MI

Telocytes (TCs), interstitial cells discovered only in 2005, have been repeatedly discussed regarding their potential for heart regeneration. TCs have been described in all the layers of the heart wall making close contact with CMs, interstitial components, as well as stem cells found in epicardial stem cell niches. One of the most essential supposed functions of TCs is the functional and morphological integration of the whole cardiac microenvironment. Therefore, TCs seem to have a great potential for cardiac regeneration and repair [153,154]. After transplantation of cardiac TCs into infarcted myocardium, the decrease in infarct size and improved myocardial function seem to result from increased angiogenesis and decreased fibrosis [155]. The exact mechanism behind TCs effect on angiogenesis was elucidated in a recent paper by Liao et al. The authors cultivated TCs in a conditioned medium and subsequently isolated TC exosomes (TCexos) for miRNA profiling. TCexo-derived miRNA-21-5p downregulated the apoptosis pathways of endothelial cells under hypoxic conditions, thus providing a suitable environment for cardiac regeneration. Based on the success of TC transplantation in animal models of MI, it can be summarized that TCs might be used in the future within cell-based and cell-free therapy alike [156]. However, most experimental studies still merely focus on elucidating the exact roles of TCs in normal conditions and pathological states, preventing us from reaching a definitive conclusion which needs to be substantiated by a more robust body of data. Nevertheless, a 2020 study provided yet another interesting finding on the integrative role and regenerative potential of TCs in the cardiac microenvironment. Using the Western clawed frog (*Xenopus tropicalis*), a species with high cardiac regenerative capacity, Lv et al. studied TCs after surgical removal of the apex of the frog heart. On Day 8, the authors observed that TCs quickly recovered and were located in close vicinity to regenerating CMs at the injured site. This finding may point to the vital role of TCs in the initiation, progression, and maintenance of cardiac regeneration. Based on such findings, it can be hypothesized that TCs may lay the regenerative foundation in terms of providing a necessary mechanical and functional base for other cells to actualize their regenerative capabilities [157]. Perhaps, the most suitable way is to see TCs not as yet another alternative cell population with regenerative potential, but as a prerequisite for the orchestration of the whole process.

## 5. Cell-Free Therapy for MI

### 5.1. Exosomes

One of the most studied approaches to cell-free therapy of MI is the use of extracellular vesicles (EVs). According to MISEV 2018 (Minimal Information for Studies of Extracellular Vesicles 2018)—a position statement of ISEV (International Society for Extracellular Vesicles), EVs is an umbrella term describing cell-released particles with different properties, e.g., size, density, biochemical composition, or biogenesis. The terms EV and that for a specific type of particle (e.g., exosome) should not be used interchangeably as is often seen in literature. However, this is a demanding task since there is a lack of consensus on specific markers of EVs subtypes [158]. Nevertheless, the first cell-free therapy for IM we are about to discuss concerns those EVs described in literature as exosomes. Exosomes have proven to be essential mediators for cellular communication and the antigen-presenting process. Moreover, they help transferring genetic information in the form of mRNAs and microRNAs (miRNA), play a crucial role in inflammatory process modulation, and influence tissue repair, stem cell maintenance, and pathological processes in various organs. These membrane-bound nanovesicles carry a cocktail of critical biological molecules, such as lipids, genetic material (mRNAs, non-coding (nc) RNAs (e.g., miRNAs), and rarely DNAs), cytoskeletal proteins, heat shock proteins, growth factors, cytokines, antigen-presenting molecules, metabolic enzymes, tetraspanins, and other signalling receptors. Several recent studies suggest that their unique cargo composition could make them a novel cell-free tool for disease diagnostics and therapy, including MI [159,160].

The therapeutic effect of EVs derived from different stem cells has been extensively studied in animal models. In general, exosomes isolated from mouse ESCs [161], BM-MSCs [162,163,164], hematopoietic stem cells (HSCs), CPCs [165], endothelial progenitor cells (EPCs) [166], adult CMs [21], CDCs [167], and CM-iPSCs [168] have proven their ability to improve heart regeneration in terms of ejection fraction, fractional shortening, reduction of infarcted size, increased angiogenesis, and decrease in oxidative stress.

It has been demonstrated that the most beneficial effect on cardiac regeneration after MI have EVs derived from MSCs (MSC-EVs) and iPSCs (iPSC-EVs). MSC-EVs have the ability to suppress CM death, enhance neovascularization, and reduce fibrosis, inflammation, and size of infarction. MSCs act on different cell types of the heart via several mechanisms, such as transfer of bioactive miRNA, long non-coding (lnc) RNA, and exosome-associated proteins. It was found that for the contraction improvement and reduction of apoptosis are responsible the high concentrations of miR-125-5p [169], and on the other side, the neovascularization is enhanced via miR-210, miR-126, miR-21, miR-23a-3p, miR-130a-3p, and miR-132 [170,171,172]. Moreover, Shen and He discovered that behind the reduced inflammation of infarcted heart tissue lies the action of exosomal miR-21-5p, which promotes the polarization of macrophages to the M2 phenotype characterized by secretion of various anti-inflammatory factors and growth factors [173]. (A detailed discussion of miRNAs as a cell-free approach is provided in a separate subsection). The study of Chen et al. further proved the positive impact of MSC-derived exosomes, which in the performed experiment preserved myocardial structure and slowed down cardiac remodeling in a mice model of cardiac hypertrophy during pressure overload [174].

**Figure 1:**
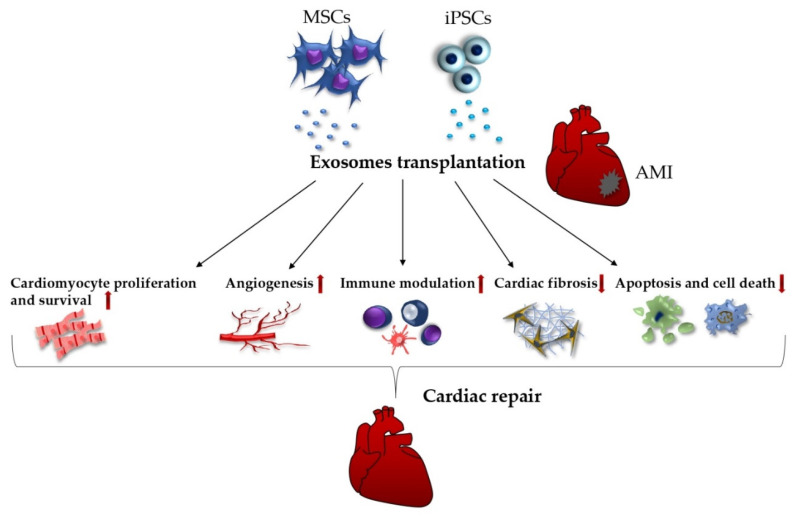
Stem cell-derived exosomes and their influence on various aspects of post-infarction myocardial repair.

Similarly, EVs derived from iPSC-CMs have a strong therapeutic potential, mainly by circumventing the challenges associated with iPSCs themselves, such as tumorigenicity or chromosome aberrations. These findings support the study of Adamiak et al., who compared the regenerative effectivity of iPSCs and iPSC-EVs in a mouse MI model. As was expected, the iPSC-EVs caused more prominent improvement in left ventricular function and perfusion, as well as apoptosis and hypertrophy amelioration over the iPSCs [168]. In addition, Kurtzwald-Josefson et al. isolated exosomes from cardiac fibroblast-iPSCs and found that they exhibit lower expression of miR-22, a vital regulator of cardiac hypertrophy and remodeling, therefore making them a future potential source for myocardial recovery after MI [175]. The promising effects of exosome transplantation on different aspects of post-MI regeneration are depicted in Figure 1.

Even though the application of exosomes is still far from clinical therapy due to several challenges (e.g., elimination of off-target effect, maximizing the cardiac regenerative effects, and generation of a highly reproducible and consistent population), they certainly bring new ideas for future MI treatment [176].

### 5.2. Non-Coding RNAs

As already mentioned, a promising therapeutic approach to the regeneration after MI is the use of non-coding (nc) RNAs (especially miRNAs), which are able to modulate different molecular and cellular processes and can be targeted with high affinity and specificity. They are involved in degradation and inhibition of translation of protein-coding genes [177]. Each miRNA can control several cellular pathways at once because they modulate 10–100 mRNA genes. Moreover, in mammalian genomes, miRNAs form clusters and are transcribed as polycistronic primary transcripts. Thus, they can regulate every aspect of cellular function, including growth, development, and cell death [178,179]. Several current studies have confirmed the role of miRNAs in the development of diseases of the cardiovascular system, including MI [180,181].

It was revealed that various miRNAs play crucial roles during MI, including regulation of CM proliferation, induction of apoptosis, and autophagy. They are also involved in the regulation of inflammation, angiogenesis, and affect myocardial fibrosis [182]. For instance, it was shown that the overexpression of miR-98 inhibited apoptosis by affecting Fas/Caspase 3 pathway thereby alleviating MI [183]. Another study showed that reduced expression of let-7a and let-7f accelerated apoptosis and led to cardiac hypertrophy and decreased ejection fraction [184]. Another proved apoptotic regulators in the circulatory system are miR-21, miR-26a, and miR-124 [185,186,187]. In addition to apoptosis, which has the task of removing damaged cells, autophagy plays an important role in cell defense that enables CMs to conserve adequate energy during MI. The most important miRNAs regulating autophagy of CMs are miR-22, miR-132, miR-206, and miR-223 [182].

Various miRNAs also significantly affect proliferation and differentiation of CMs [188]. Gabisonia et al. demonstrated that overexpression of miR-199a restored the proliferation and differentiation of CMs and thus enhanced heart function after MI in pigs [189]. Another study revealed that an miR-17-92 cluster induces sufficient proliferation of CMs in postnatal and adult hearts and was considered a promising target for cardiac regeneration and repair [190]. MiR-302–367 cluster is another group of miRNAs that have a regulatory effect on heart regeneration by promoting CM proliferation through regulation of the Hippo signalling pathway [191]. Further study showed that miR-128 inhibition in the heart of adult mice improved heart regeneration and mitigated cardiac dysfunction, which was linked to the governing effect of miR-128 on the chromatin regulator (SUZ12), further improving cell cycle activity [192].

Various miRNAs also initiate and regulate inflammatory response in infarcted heart. For instance, overexpression of miR-26b improved myocardial remodeling caused by MI in mice by suppression of the MAPK pathway through binding to prostaglandin endoperoxide synthase 2 [193]. In contrast, Icli et al. have shown that miR-26a downregulation improved the heart function of MI mice, which was linked to the inhibitory effect of miR-26a on angiogenesis [194]. Further research demonstrated that antagonistic miR-375 exerted a shielding effect on the heart by inducing angiogenesis, reducing inflammation, and inhibiting apoptosis [195]. Moreover, Wang et al. in a recent study, showed that upregulation of miR-335 reduced MI damage by reducing oxidative stress, cell death and inflammation [196].

Vascular regeneration is another significant process influencing regeneration after MI. In addition, in this case, several miRNAs play pivotal role. For instance, miR-210 and miR-21-5p endorsed vascular regeneration through different pathways. Qiao et al. demonstrated that miR-21-5p supported angiogenesis and survival of CMs through the phosphatase and tensin homolog/Akt pathway [197]. Another research study showed that endogenous reduction of miR-185-5p in endothelial cells induced by hypoxia increased *CatK* gene expression and led to angiogenesis induction and thus sped up recovery of cardiac function after MI [198].

The effect of miRNAs on fibrosis after MI has attracted the attention of many research teams. Jazbutyte et al. reported that increased levels of miR-22 supported cardiac fibroblasts proliferation and thus promoted fibrotic process [199]. Other research group demonstrated that miR-21 has an important effect in the process of cardiac fibroblast activation and cardiac fibrosis after MI through the TGF-β/Smad7 signalling pathway [200]. Moreover, a clinical study performed by Gupta et al. provided evidence that the circulating levels of miR-22 in patients with heart failure were positively correlated with their mortality. This highlights miR-22 as a promising therapeutic and biomarker candidate for cardiovascular disorders [201].

Myocardial remodeling includes the enlargement of the ventricular cavity, progressive hypofunction, collagen storage outside myocardial cells, inflammatory cell infiltration, and progressive cell death. Some miRNAs (e.g., miR-130a, miR-144, miR-181) regulate phenotypes related to ventricular remodeling through downregulation of the expression of certain genes, so they can be regarded as potential targets for the prevention of deleterious ventricular remodeling after MI [202,203,204].

In addition to miRNAs, there are some other ncRNAs, such as lncRNAs and circular (circ) RNAs, which also play a crucial role in the pathogenesis of MI. It was shown that some lncRNAs can modulate miRNAs to affect the target processes in both detrimental and beneficial way [205,206]. In many cases, one lncRNA can interact with multiple miRNAs and thus inhibit their function, and then exert different physiological and pathological regulatory effects [207]. Similar to lncRNAs, circRNAs also interact with miRNAs during MI, thereby reducing/aggravating MI [208,209].

### 5.3. Gene Therapy

Another promising therapeutic option in the treatment of MI is gene therapy. Understanding of the molecular mechanisms in MI gradually led to identification of novel targets that are difficult to influence pharmacologically, but can be altered by gene therapy. The most promising approach suitable for MI treatment is based on adeno-associated viral (AAV) vectors in combination with several therapeutic targets (e.g., inhibition of GRK2, restoring of SERCA2a function, phosphorylation of phospholamban, upregulation of VEGF, etc.) [210,211]. Recently, several clinical trials testing the efficacy of AAV vectors have been launched and are ongoing [212,213,214]. Their results may support the translation of this approach into clinical practice. However, there are still many challenges to overcome, including low gene transduction efficacy, expensive and demanding production of vectors, or the issue of minimizing the total vector dose for the purpose of cost reduction, etc. [212].

### 5.4. Acellular Cardiac Patches

We have already discussed the use of cardiac patches as a promising approach in cell-based therapies; however, it is worth mentioning the importance of acellular patches, which have been evaluated as mechanical–structural support for MI. Acellular patches possess several advantages over cellular patches because of their off-the-shelf availability for immediate implantation, extended shelf lives, better mechanical and functional properties, and limited immune reactions [215]. In the work of Serpooshan et al., an engineered acellular collagen patch was transplanted into the infarcted myocardium of adult murine hearts. The physiological outcomes were evaluated 4 weeks post-infarction and compared with the control group. According to the analyses, patched hearts integrated with host native cells (fibroblasts, smooth muscle cells, epicardial cells, and immature CMs), therefore, preserving contractility, reducing left ventricular remodeling, and suppressing fibrosis [216]. Shah et al., examined the therapeutic effect of acellular cardiac patches derived from decellularized porcine myocardium. The patches of two different thicknesses (300 and 600 µm) were grafted to the infarcted area of the rat myocardium. After implantation, the authors observed firm attachment of cardiac slices to the host myocardium and robust cellular infiltration with notably higher density of M2 macrophages, as well as significant neoangiogenesis [217]. Moreover, there was a significant improvement in LVEF and fractional shortening compared to the control group. Huang et al., recently published their results of the generation of a fully acellular artificial cardiac patch composed of decellularized porcine myocardial ECM scaffold and synthetic cardiac stromal cells [218]. The potency of such a patch construct was examined on rat and porcine models of AMI. Among the encouraging results reported by authors were reduced scarring, increased angiomyogenesis, and improved cardiac function resulting in overall cardiac recovery. In summary, the acellular cardiac patches indeed bear therapeutic benefits; however, more studies need to be conducted on large animal models with longer follow-up.

## 6. Summary and Possible Prospects

Currently, treatments of MI are mostly only cardioprotective, focusing on protecting myocardial tissue from cell death and delaying the progress of heart failure; however, they cannot regenerate infarcted myocardial tissue [219]. For that reason, research interests worldwide have focused on finding the best way to repair the damaged tissue either by stimulating its endogenous regenerative capacity or generating new cells and bioengineered tissue as a replacement. A better understanding of molecular and cellular mechanisms of underlying heart regeneration contributes to the rapid development of bioengineered strategies based on cellular (MSCs, CPCs, EPCs, HSCs, or iPSCs) and acellular particles (EVs) in combination with various biomaterials. Lately, patching up the damaged heart area using stem cell-based therapies seems highly efficient.

On top of that, the invention of patient-specific iPSCs, which are efficiently differentiated into iPSC-CMs, has further improved the construction of human cardiac muscle patches. However, challenges hampering stem-cell-based therapies, such as low cell engraftment rate, short survival time, mediate maturation, and poor coupling success, often result in arrhythmia post-transplantation. Therefore, there is a need to ameliorate and optimize these methods.

On the other hand, the emerging concept of cell-free therapy has sparked the enthusiasm of the broad research community. It has been shown that cargo material carried by EVs significantly improves cardiac histology, function, cell proliferation, and tissue remodeling. Moreover, existing drawbacks related to the use of EVs in clinical practice are now being solved by bioengineered modifications focusing on increasing targeting ability and accessible EVs delivery [220]. Last but not least, a recently discovered cell population—telocytes, found in all parts of the heart wall—should be considered an alternative cell source reliable for cardiac regeneration, thanks to their contribution to cardiac physiology and regenerative response to heart injury.

Despite the progress of conventional therapies, which involve pharmacological agents (anticoagulants, antiplatelets), and interventional therapies (PCI, CABG), the overall therapeutic effect on injured myocardium is poor, resulting in a pressing need for alternative approaches to enhance heart tissue regeneration and restore its functionality. All in all, each treatment method, whether it is the currently used (conventional), or promising cell-based and cell-free therapy, has its pros and cons.

The advantages of conventional therapies include:

- Many years of experience with clinical application;
- Successful mitigation of mortality rates;
- Refined and elaborated guidelines available for all clinicians.

The disadvantages of conventional therapies include:

- Only moderate effect in addressing the post AMI complications;
- Impossibility to suppress the loss of functional heart muscle;
- Daily need of medications;
- High risk of recurrent development of the heart failure;
- Shortage of donor hearts.

The advantages of cell-based therapies and tissue engineering include:

- Patient specificity;
- High cardiac differentiation potential;
- Elimination of immune rejection;
- Development of large-scale cultivation systems;
- Substantial progress in understanding the molecular and cellular mechanism of MI;
- Encouraging results of iPSC-CMs regenerative capacity in animal models;
- Use of stem cells with additional 3D cardiac engineered biomaterials;
- Transplantation of iPSC-CMs as a patch or sheet resembling the structure and function of native myocardium, and restoring the lost function of damaged myocardium.

The disadvantages of cell-based therapies and tissue engineering include:

- Lack of uniform cardiac differentiation protocols;
- Incomplete stem cell differentiation into mature and functional CMs;
- Safety issues regarding the possible tumor formation caused by reprogramming factors, and epigenetic abnormalities;
- Heterogeneity of iPSCs populations;
- Costly and time-consuming process of iPSC-CMs generation;
- Lack of control over transplanted cell population;
- Poor graft survival rate;
- Difficulties in vascularized cardiac patches integration with host tissue regarding to electrical, mechanical, vascular, and biochemical compatibility;
- Possible toxicity of various biomaterials’ nanoparticles;
- Post-transplantation arrhythmia due to lack of electromechanical coupling with host cells;
- Clinically moderate benefits;
- Need for human clinical trials.

The advantages of cell-free therapies include:

- Beneficial roles of EVs paracrine bioactive components in terms of protection of heart tissue from disease progression (promotion of angiogenesis, inhibition of ventricular remodeling, improvement of heart function, inhibition of local inflammation, regulation of immune responses);
- Reduction of above-mentioned challenges related to the use of cell-based therapy;
- Positive therapeutic effect demonstrated in animal studies;
- Possibility to generate bioengineered exosomes with enhanced targeting properties;
- Low/no risk of mutagenesis.

The disadvantages of cell-free therapies include:

- Do not fulfil the medical need for heart regeneration in MI patients;
- Need for clear understanding of the paracrine signalling pathways;
- Issues such as lack of targeting, and low retention related to direct infusions of EVs paracrine agents;
- Need for establishment of standardized fabrication and purification methods;
- Challenging quality control of cell-free products;
- EV immunogenicity;
- Problems with biosafety, and biodegradation of bioengineered products;
- Significant reduction of retained growth factors within acellular cardiac patches;
- Need for human clinical trials.

Taken together, we can conclude that despite the significant number of pitfalls related to cell-based therapies, their successful combination with tissue engineering approaches and gene-editing technologies may result in construction of human functional myocardial tissue which would undoubtedly represent a therapeutic alternative for millions of patients suffering from MI worldwide. Admittedly, desired clinical outcomes may become reality only after resolving all mentioned issues. Unfortunately, this will be a very demanding and time-consuming task which requires a joint effort of many research teams around the globe. Nevertheless, we hope that the clinical application will be accomplished within a few years. The use of acellular cardiac patches as a part of cell-free therapy has shown positive outcomes, but their application must be further examined. On the other hand, cell-free therapy will most likely become a successful supportive approach in the heart regeneration process as a specific form of drug therapy.

## Figures and Tables

**Table 1:** Overview of selected clinical studies on cell-based therapy.

Clinical Trial	Phase	Type of Stem Cell	Rout of Delivery	Status	Primary Outcome Measures	Results
BOOST (NCT00224536) [87]	1	(Bone marrow-derived mesenchymal stem cells (BM-MSCs)	Intracoronary injection	Completed 2003	- change in global (left ventricular ejection fraction) LVEF	- no long-term benefit on left ventricular (LV) systolic function - acceleration of LVEF recovery
BM-MSCs transfer in AMI (NCT00264316) [88]	2	BM-MSCs	Intracoronary injection	Completed 2005	- increase in global LVEF	- no augmentation of LVEF recovery
MYSTAR Study (NCT00384982) [89]	2	BM-MSCs	Intracoronary/or combination of intracoronary with percutaneous intramyocardial	Completed 2008	- changes in resting myocardial perfusion defect size - Changes in global LVEF	- improvement of cardiac function - significant increase in right ventricular ejection fraction (RVEF)
Stem Cell Therapy to Improve Myocardial Function in Patients with AMI (NCT00316381) [90]	N/A	CD34^+^/CXCR4^+^/C-met stem cells	Intracoronary injection	Completed 2008	- LVEF and volumes measured by echocardiography and angiography	- positive correlation between the mobilization of CD34^+^/CXCR4^+^/C-met stem cells into peripheral blood and LVEF
TRACIA study (NCT00725738) [91]	2/3	BM-MSCs	Intracoronary injection	Completed 2009	- evaluation of LVEF increase between stem cell group and control group	No effect on LV function
SEED-MSC (NCT01392105) [92]	2/3	BM-MSCs	Intracoronary injection	Completed 2010	- absolute changes in global LVEF	- improvement in the LVEF
Bmmsct (NCT04421274) [93]	2/3	BM-MSCs	Percutaneous coronary injection	Completed 2011	- changes in myocardial metabolic activity - change in LVEF	- no effect on LVEF and myocardial viability
The late TIME Study (NCT00684060) [94]	2	Bone marrow (BM)- mononuclear stem cells	Intracoronary injection	Completed 2012	- change in global LVEF - regional LVEF	- no improvement of LVEF
WJ-MSC-AMI (NCT01291329) [95]	2	WJ-MSCs	Intracoronary injection	Completed 2012	- quantitative myocardial metabolic and perfusion - safety and efficacy	- significantly greater absolute increase in the myocardial viability and perfusion
EMRTCC (NCT00350766) [96]	2/3	BM- mononuclear stem cells	Intracoronary injection	Completed 2014	- change in global LVEF	- no improvement of the echocardiographic parameters of systolic function
HUC-HEART (NCT02323477) [97]	1/2	(Umbilical cord stroma) UCS- MSCs	Intramyocardial injection	Completed 2018	- ventricular remodeling	- possible positive effect in scar tissue reduction and restoration of ventricular wall function
REGEN-AMI (NCT00765453) [98]	-	BM- progenitor cells	Intracoronary injection	Completed 2018	- longitudinal change in LVEF	- slight non-significant improvement in LVEF
ALLSTAR (NCT01458405) [99]	1/2	Allogeneic Cardiosphere-Derived Cells	Intracoronary injection	Completed 2019	- safety and effective decreasing of infarct size	- no scar size reduction - significant reduction in LV end-systolic volume
Stem cells in acute myocardial infection (AMI) (NCT04340609) [76]	1/2	Umbilical cord (UC)-MSCs	intravenous injection and intracoronary injection	Completed 2022	- major adverse cardiac events endpoints of mortality - re-infarction - target vessel revascularization - heart failure hospitalization	- final data collection
Heart Patch for MI COVID-19 (NCT04728906) [100]	N/A	- patch seeded with amnion epithelial stem cells and autologous cardiomyocytes (CMs)	- patch transplantation during coronary artery bypass grafting surgery	Recruiting	- change of the ischemic burden - Change in the regional heart wall motion abnormality	- expected completion date is September 2022
LAPiS Study (NCT04945018) [101]	1/2	Induced pluripotent stem cells (iPSCs)	HS-001-D needle transplantation	ongoing	Evaluation of safety and tolerability of iPSC-derived CM spheroids	- expected completion date is March 2024
